# Collaborative design of a care pathway for pharmacy-based PrEP delivery in Nigeria: insights from stakeholder consultation

**DOI:** 10.1186/s12913-024-12107-4

**Published:** 2024-12-18

**Authors:** Obinna I. Ekwunife, Theodora C. Omenoba, Ugochi Eyong, Valentine Okelu, Michael Alagbile, Ifeanyi Ume, Ambrose Eze, Aderinola Fisayo, Gloria Aidoo-Frimpong, Farah Shroff, Chimezie Anyakora

**Affiliations:** 1 Division of Population Health, Department of Medicine, University at Buffalo, New York, 14203 USA; 2 Department of Clinical Pharmacy and Pharmacy Management, Veritas University, Abuja, Nigeria; 3 Bloom Public Health, Abuja, Nigeria; 4 Department of Clinical Pharmacy and Pharmacy Management, Nnamdi Azikiwe University, Agulu, Nigeria; 5 Society for Family Health, Abuja, Nigeria; 6 Association of Community Pharmacists of Nigeria, Lagos, Nigeria; 7 Pharmacy Council of Nigeria, Abuja, Nigeria; 8 Department of Epidemiology and Environmental Health, University at Buffalo, New York, USA; 9 Faculty of Medicine, The University of British Columbia, Vancouver, Canada; 10 School of Science and Technology, Pan-Atlantic University, Lagos, Nigeria

**Keywords:** Stakeholder analysis, Differentiated service delivery, HIV prevention, Preexposure prophylaxis, Nigeria, Community pharmacies, Africa

## Abstract

**Background:**

HIV remains a significant public health problem, particularly in Africa, where two-thirds of global cases occur. Nigeria is among the three countries with the highest burden. Despite free access to pre- and post-exposure prophylaxis (PrEP and PEP) in Nigerian hospitals, stigma, distance, and restrictive clinic hours hinder uptake, especially among vulnerable populations. Building on the successful pilot implementation of pharmacy-based PrEP delivery in Kenya, we engaged Nigerian stakeholders in adapting the model, addressing user and provider concerns to ensure effective implementation in Nigeria.

**Methods:**

The stakeholder meeting took place in Abuja, Nigeria, which is selected for its central location and accessibility to various stakeholders, particularly those involved in HIV prevention efforts. The participants were purposefully selected to ensure diverse representations, including youth who are potential PrEP users, pharmacy providers, regulators, and representatives from civil society organizations. The meeting utilized the Nominal Group Technique (NGT)—a structured method for facilitating group decision-making and prioritizing ideas—to adapt the Kenyan pharmacy-delivered PrEP model for implementation in the Nigerian context. Mock role play was conducted to help participants understand the care pathway. The discussions culminated in identifying challenges and viable strategies for implementing the model in Nigeria.

**Results:**

The one-day stakeholder meeting on 9 October 2024 was attended by 20 participants from various sectors involved in HIV prevention services. Stakeholders expressed enthusiasm for pharmacy-based PrEP delivery while acknowledging challenges associated with clinic-based services, such as stigma, limited hours, and long wait times. The key recommendations included training pharmacy providers, increasing awareness, ensuring confidentiality, establishing referral linkages, and integrating program data into the Health Management Information System (HMIS) as well as ensuring commodity availability and access. To enhance the success of the pilot study, stakeholders proposed engaging a research assistant, forming a monitoring team, and submitting the results to the Pharmacy Council of Nigeria (PCN) for review.

**Conclusions:**

The identified challenges and strategies for implementing the model in Nigeria will inform the development of a refined pharmacy-delivered PrEP framework that is ready for pilot testing and potential scaling across the country.

## Introduction

Globally, HIV affects approximately 40 million people, with two-thirds of the cases occurring in Africa [1]. Nigeria, one of the top three countries with the highest HIV burden, bears a substantial share of new infections, vertically transmitted infections, and AIDS-related mortality [2]. In 2019, young adults in Nigeria aged 15–24 years accounted for the highest number of new infections among all age groups, accounting for nearly one-third of all new HIV cases [3].

Despite the availability of effective HIV prevention interventions, such as pre- and post-exposure prophylaxis (PrEP and PEP) at no cost in Nigerian HIV treatment hospitals, numerous social, structural, and logistical barriers persist [4–6]. Barriers, including stigma, geographic distance from healthcare facilities, and restrictive clinic hours, limit access to and uptake of these essential services among Nigeria’s most vulnerable populations [6, 7]. Additionally, limited access to comprehensive sexual education and prevalent risk behaviors exacerbate these barriers, contributing to persistently high rates of new HIV infections among youth, which remains a significant concern [3]. Recognizing this challenge, Nigeria’s Ministry of Health has identified the expansion of PrEP access as a priority for young people, who face a greater risk of HIV acquisition than other groups do [3]. Various studies have shown that PrEP and PEP, when taken as prescribed, are effective in reducing the risk of acquiring HIV, especially for those at high risk [8–11].

Although current PrEP delivery strategies in Nigeria prioritize groups such as serodiscordant couples, sex workers, people who inject drugs, and men who have sex with men through one-stop shops, these strategies often overlook at-risk youth [3]. In Nigeria, adolescents and young adults are at increased risk of HIV acquisition, yet no tailored PrEP delivery strategy exists for this group [3]. To address this gap, expanding access to PrEP through task-shifting strategies that engage at-risk young people outside of traditional hospital settings is needed. Linking PrEP services to locations where young people seek sexual and reproductive health products, such as retail pharmacies, efficiently reaches at-risk young individuals [12]. However, this must be supported with providers’ training, adequate supervision and commodity availability. Community pharmacies are increasingly seen as promising strategies for PrEP delivery. Previous studies in Nigeria have shown that private community pharmacies and proprietary and patent medicine vendors (PPMVs) play important roles in providing sexual and reproductive health services to young people [13–15]. This suggests that pharmacies could offer a similarly discreet and accessible option for PrEP delivery, helping to overcome significant barriers such as stigma and confidentiality concerns associated with hospital PrEP delivery methods [12, 16]. Retail pharmacies are numerous in Nigeria and are often located in high-traffic areas, making them convenient access points for PrEP [17]. Additionally, other studies have demonstrated that integrating community pharmacies as centers for antiretroviral therapy delivery is feasible and can lead to high retention in care and low loss-to-follow-up rates [18–20].

Delivery models that involve private pharmacy providers and/or support from nurse navigators have been successfully pilot tested in Kenya [12, 16]. However, their acceptability, feasibility, and implementation costs are yet to be demonstrated in Nigeria and other sub-Saharan African countries. Building on the successful implementation of PrEP in Kenya, we aim to explore the potential application of a similar model in Nigeria. To do so, we must first adapt the Kenyan pharmacy-delivered PrEP model to the Nigerian context to create a tailored care pathway for pharmacy-based PrEP delivery. Therefore, we convened stakeholders to review and adapt the Kenyan model, addressing the concerns of potential users, providers, and other key stakeholders, to ensure successful implementation. This paper presents the findings from our collaborative effort to develop a context-specific pharmacy-based PrEP delivery model in Nigeria.

## Methods

### Consensus building approach

We employed the Nominal Group Technique (NGT) to achieve consensus among diverse stakeholders on the challenges and viable strategies for implementing the Kenyan pharmacy-delivered PrEP model in Nigeria. NGT is a structured method used to facilitate group decision-making and prioritize ideas [21]. This technique has been widely utilized in designing HIV prevention interventions [22–25]. The process involved two steps. First, participants working in small groups listed potential challenges associated with implementing the model and proposed solutions, categorizing them as either requiring immediate or long-term actions. Next, the ideas were aggregated as the small groups were reconvened, with similar ideas merged. The participants reached a general agreement on the most important ideas, which were then documented.

### Study setting and design

The one-day stakeholder meeting was held on 9 October 2024 in Abuja, Federal Capital Territory, Nigeria. Abuja was chosen for its central location and accessibility to stakeholders given that it is the seat of government and accessible to stakeholders from various regions. Additionally, among the seven U.S. President’s Emergency Plan for AIDs Relief (PEPFAR) priority states in Nigeria, Abuja ranks fourth in the number of key population hotspots [26].

### Stakeholder selection

The participants for the stakeholder meeting were purposively selected to ensure diverse representations across sectors. They included youth representatives, aged 18–25, who are potential PrEP users and were identified through pharmacies selling sexual and reproductive health products; pharmacy providers; pharmacy regulators; and representatives from regulatory bodies and civil society organizations. Among the pharmacy providers, we invited two whose pharmacies would participate in the pilot implementation of the pharmacy-based PrEP model, the next phase following the stakeholder meeting. These two retail pharmacies are in distinct areas within the Abuja Municipal Area Council—one in an affluent neighborhood and the other in a less privileged area. This geographical contrast enables us to capture a diverse range of clients across various socioeconomic and demographic groups. For regulatory and civil society representatives, participants were drawn from organizations such as the Pharmacy Council of Nigeria (PCN), the Association of Community Pharmacy in Nigeria (ACPN) and nongovernmental organizations involved in HIV control, such as the Society for Family Health.

### Meeting activities

**Fig. 1:**
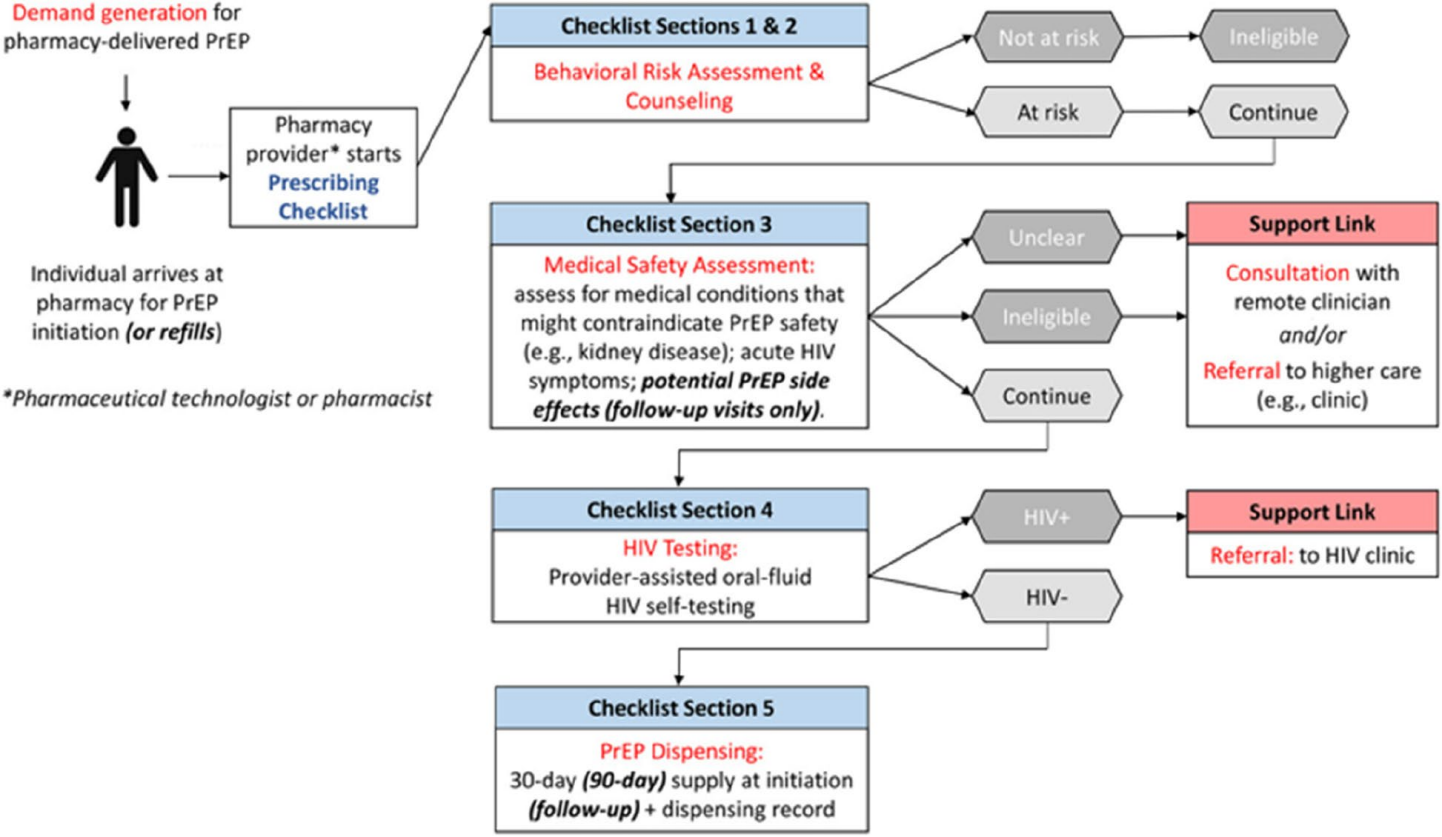
Care pathway for pharmacy-delivered oral PrEP services used in Kenya. Copied from Journal of the International AIDS Society, Volume: 26, Issue: 6, First published: 12 June 2023, DOI: (10.1002/jia2.26131)

The one-day stakeholder meeting began with an overview of its purpose and objectives, presented by a representative from Bloom Public Health, who highlighted the role of PrEP in Nigeria’s HIV prevention strategy. A member of the research team, who had been involved in the pilot studies in Kenya, then introduced the Kenyan pharmacy-based PrEP delivery model and shared key findings from its pilot implementation (Fig. 1). In the Kenyan model, trained providers offer PrEP and PEP services to eligible clients via a prescribing checklist under the supervision of a remote clinician [16, 27]. Clients who do not meet the checklist criteria (e.g., those with a history of kidney disease) are referred to a nearby public clinic for further eligibility assessment and in-person clinical care. The participants engaged in a mock role-playing of the care pathway via the prescribing checklist, which covers key components such as screening, medication safety assessment, counselling, HIV testing, prescribing, and dispensing. This was then followed by a break-out session for small group discussion (1 h), where participants were divided into four groups, with the members of the research team serving as facilitators to oversee the session, ensuring equitable participation among all stakeholders. Each group independently generated ideas on potential challenges and solutions for implementing the Kenyan model in Nigeria, utilizing structured worksheets to identify challenges and potential solutions (Appendix 1). Each group discussed, categorized, and prioritized the identified solutions. Problems were categorized as those requiring immediate versus long-term solutions. After the breakout session, all participants reconvened to collectively agree through consensus or vote on the most important challenges and strategies. The prioritized challenges and strategies were then compiled and summarized. Meeting proceedings, detailed notes of the discussions, NGT results, and prioritized strategies were documented. A summary report of the meeting was prepared and shared with stakeholders for feedback and validation.

## Results

### Meeting attendance

The meeting included 20 stakeholders from the Pharmacy Council of Nigeria (PCN) (*n* = 1), the Association of Community Pharmacists of Nigeria (ACPN) (*n* = 1), Civil Society Organizations and health professionals (*n* = 5), pharmacy providers (*n* = 6) and youth representatives (*n* = 7).

### Identifying and addressing implementation challenges

Stakeholders were enthusiastic about the project and acknowledged the shortcomings of clinic-based PrEP delivery, such as stigma, provider attitudes, limited hours of operation, long waiting times, travel distance and cost, and emphasized their support for Pharmacy-based PrEP delivery.

Stakeholders identified several challenges and solutions during both small and large group discussions regarding the pilot implementation of pharmacy-based PrEP delivery and its broader scale-up in Nigeria. They recorded their observations via a worksheet (see Appendix 1) provided as a guide for documentation. These findings are summarized in Table 1. The proposed solutions are categorized into short-term solutions, which can be implemented during the pilot study, and long-term solutions, which are applicable to future scale-up efforts.

**Table 1:** Potential challenges and solutions to pharmacy-based PrEP delivery

Challenges	Short term solution	Long term solution
*Demand Generation*		
Limited awareness of community pharmacies as delivery points for oral PrEP	• Raise awareness through community volunteers and social media campaigns • Develop and distribute tailored IEC materials (leaflets) • Use branding and signage to highlight service providers • Engage students in tertiary institutions via social media ads and dedicated outreach programs	• Integrate pharmacy-based PrEP delivery into the National Health Promotion Policy (NHPP) by partnering with the relevant agency • Share generated evidence with key stakeholders by presenting findings at PSN and ACPN conferences, and by highlighting key policy implications to the Pharmacy Council of Nigeria for review by its governing body
Concerns about the stigma associated with obtaining oral PrEP from pharmacies	• Train providers on stigma reduction and the importance of confidentiality • Counselling should inform clients about the possibility of consultation with a remote clinician	
*Assessment*		
Insufficient capacity to engage with high-risk HIV clients in pharmacies	• Train pharmacy providers on patient counselling skills	• Integrate pharmacy-based PrEP delivery training in Mandatory Continuing Professional Development (MCPD)
Some pharmacy premises lack the necessary infrastructure to ensure client confidentiality	• Only pharmacies adhering to PCN guidelines on having consultation room will be engaged in service delivery • Interested pharmacies without consultation room(s) should create one to be eligible	
Lack of time to go through the assessment checklist	• Rescheduling of some clients to off-peak period • Consistent use of the assessment checklist, leading to mastery of the tool and improved delivery efficiency	• Using e-platforms for pharmacy-based PrEP delivery
Non existing referral linkage between community pharmacies and healthcare facilities	• Create a referral directory that would comprise all health facilities providing HIV/PrEP services within the geographic location of the pharmacy • Engage referral health facilities to inform them about the pharmacy's referral process and secure their support and collaboration	• Establish interoperability between the community pharmacy EMR and health facility EMR systems
*Testing*		
The cost of HIV testing services in private pharmacies may be prohibitively high	• Implement controlled pricing that is mutually agreed upon by providers and accessible for most pharmacy clients	
Last-mile delivery of testing kits from the central store or PHC to community pharmacies may place added strain on pharmacy providers		• Include community pharmacies in the master list of health centers designated to receive supplies from the central store • Utilize distributors, such as wholesalers or NGOs, to procure supplies from central stores and distribute them to pharmacies at a controlled price
Disruptions may arise in the consistent supply of kits for the project	• Refer clients to health facilities when there is stock out	
Pharmacy providers may be unfamiliar with the proper disposal procedures for sharps and used HIV test kits	• Provide training to pharmacy providers on standard waste management procedures and supply them with biohazard boxes	• Link participating pharmacies with primary health centers that have incinerators
Medical and laboratory scientists may oppose HIV testing in private pharmacies	• The revised testing guidelines now allow for rapid diagnostic testing in pharmacies	• Advocacy to the Medical Laboratory Science Council of Nigeria (MLSCN) to secure their support
*Dispensing*		
Pharmacy providers not authorized to initiate PrEP prescription	• Seek a waiver allowing pharmacies to initiate PrEP prescriptions for pilot studies	• Advocacy to the Federal Ministry of Health and the National Council on Health to authorize pharmacy providers to initiate PrEP prescriptions
Nonadherence to PrEP is a significant concern, particularly among long-term users	• Pharmacy providers should counsel and follow-up clients initiated to PrEP • Pharmacy providers should evaluate self-reported adherence for clients continuing PrEP and provide guidance to those who are nonadherent • Pharmacy providers must also monitor for any adverse reactions and refer clients experiencing such reactions to health centers	
*Consultation*		
Clinicians may be unresponsive when consulted by pharmacy providers	• Establish a collaborative practice agreement with selected doctors to formalize the pharmacy's operations before the launch of services	
*Operations*		
Some pharmacy providers may lack the time to attend training or may miss it altogether	• Offer both in-person and virtual training options	• Incorporate training sessions into ACPN meetings, which are widely attended by pharmacists, to facilitate scale-up
Trained pharmacists in a particular pharmacy premise may occasionally be unavailable	• Train all eligible pharmacy staff, not just pharmacists, to ensure continuity of service delivery	
*Research*		
Limited research experience among pharmacy providers may lead to inconsistent reporting practices and noncompliance with research protocol	• Engage a Research Assistant during the pilot study to ensure accurate and high-quality data reporting • Establish a monitoring team composed of some of the stakeholders to oversee the study's progress and ensure its success	• Collaborate with the Director of Pharmaceutical Services (DPS) to integrate all pharmacies providing PrEP services into the State Integrated Supportive Supervision Visits (ISSV) team • Ensure inclusion of the program data from pharmacies into Health Management Information System (HMIS) for the private sector currently under development • Submit the final project results to the Registrar of the Pharmacy Council of Nigeria (PCN)

*NHPP *National Health Promotion Policy, *PrEP* Preexposure prophylaxis, *IEC* Information, Education and Communication, *PSN* Pharmaceutical Society of Nigeria, *ACPN* Association of Community Pharmacists of Nigeria, *EMR* Electronic Medical Record, *NGO* Non-Governmental Organization, *ISSV* Integrated supportive supervision visits, *HMIS* Health Management Information Systems, *DPS* Director of Pharmaceutical Services, *MLSCN* Medical Laboratory Science Council of Nigeria

### Demand generation concerns

A major challenge related to demand generation was the low public awareness of community pharmacies being used as service delivery points for PrEP. To address this, they proposed several short-term solutions, including engaging community volunteers, running social media campaigns, distributing contextualized leaflets, using branding and signage to identify participating pharmacies, and conducting targeted outreach to high-risk youth in tertiary institutions. For long-term scale-up, the stakeholders recommended integrating messaging on pharmacy-based PrEP delivery into the National Health Promotion Policy and sharing evidence with key stakeholders through platforms such as the National HIV Prevention Technical Working Group and the PSN and ACPN conferences. They also raised concerns about the potential stigma associated with obtaining oral PrEP from pharmacies. To mitigate this, they suggested pharmacy provider training focused on confidentiality and stigma reduction.

### Eligibility assessment and confidentiality concerns

The stakeholders identified challenges regarding the assessment of clients to identify those eligible for PrEP in the pharmacy. One major issue is the insufficient capacity of pharmacy providers to engage high-risk HIV clients. To address this, they suggested that pharmacy providers should receive training on patient counselling skills, and pharmacy-based PrEP delivery training should be integrated into the Mandatory Continuing Professional Development (MCPD) program for pharmacists in the long term. Another challenge is the lack of infrastructure to ensure client confidentiality. To ensure quality service, the stakeholders suggested that only pharmacies that comply with the Pharmacy Council of Nigeria (PCN) guidelines requiring consultation rooms are involved in service delivery. Pharmacies interested in participating but lacking consultation rooms must establish one to become eligible. Additionally, the stakeholders noted that providers may struggle to find time to complete the assessment checklist. To mitigate this, they suggested that providers could advise clients to visit during off-peak periods, and in the long term, e-platforms should be explored to streamline pharmacy-based PrEP delivery. Finally, the stakeholders noted that a lack of referral linkages between community pharmacies and healthcare facilities presents a significant barrier to seamless service delivery. To address this, they suggested the development of a manual or digitized referral directory depending on the practice setting, listing all health facilities within the pharmacy’s geographic area that provide HIV and PrEP services. These healthcare facilities will be engaged and oriented to inform them about the referral process and secure their collaboration and support for accepting clients referred from the pharmacy.

### Testing services and logistics

With respect to testing for HIV in private pharmacies, the stakeholders acknowledged that the cost of testing services may be high. To address this, they suggested implementing controlled pricing that is affordable for most pharmacy clients. Additionally, they noted that ensuring the last-mile delivery of testing kits from central stores or primary healthcare centers to community pharmacies is essential. To make delivery seamless, they suggested integrating community pharmacies into the master list of health centers eligible to receive public (free) HIV supplies from the central store. Additionally, they suggested that distributors, including wholesalers or NGOs, could be engaged to procure kits from central stores and distribute them to pharmacies at a controlled price. To maintain a continuous supply of testing kits for the project, the stakeholders suggested that clients should be referred to healthcare facilities if stockouts occur in pharmacies. Additionally, they suggested prioritizing waste management by training pharmacy providers on standard waste disposal procedures and supplying biohazard boxes. Finally, the stakeholders noted that concerns from medical and laboratory scientists regarding HIV testing in pharmacies have been addressed, as the revised testing guidelines now permit rapid diagnostic testing in pharmacy settings. However, they noted that there is also the need to advocate and engage the Medical Laboratory Science Council of Nigeria (MLSCN) to secure their support.

### Dispensing and adherence support for PrEP

The stakeholders noted that dispensing PrEP in pharmacies faces several challenges. Currently, pharmacy providers are not authorized to initiate PrEP prescriptions. To address this, they emphasized the need to seek a waiver allowing pharmacies to initiate PrEP prescriptions for pilot studies, as well as to advocate for broader authorization for pharmacy providers to prescribe PrEP. Nonadherence to PrEP was noted as a significant concern, particularly among long-term users. To address this issue, the stakeholders suggested that pharmacy providers should offer counselling and follow-up (including the use of an automated reminder system) for clients who have initiated PrEP. Additionally, they should assess self-reported adherence for clients continuing PrEP and provide support to those who are nonadherent. Furthermore, they recommended that pharmacy providers monitor clients for any adverse reactions to the medication and ensure that those experiencing such reactions are documented and referred to health centers for appropriate care.

### Establishing collaborative consultation processes

For the consultation process, the stakeholders noted that pharmacy providers may encounter unresponsiveness from clinicians when seeking guidance. To address this challenge, they suggested establishing a collaborative practice agreement with selected doctors. This agreement will help formalize the pharmacy's operations and ensure effective communication before the launch of service.

### Training and availability of pharmacy staff

To address operational challenges, the stakeholders noted that some pharmacy providers may lack the time and adequate manpower to attend training or may miss it altogether. To mitigate this issue, they recommended offering both in-person and virtual training options. Additionally, incorporating training sessions into ACPN meetings—events that are widely attended by pharmacists—can facilitate the scale-up of training efforts. The stakeholders noted that trained pharmacists at specific pharmacy locations may occasionally be unavailable. To ensure the continuity of service delivery, it was suggested that all eligible pharmacy providers be trained, not just pharmacists. However, the superintendent pharmacist still bears liability for all activities carried out at the pharmacy. This comprehensive approach will help maintain quality, consistency, and effective service provision.

### Research and monitoring during the pilot study

With respect to the pilot studies to establish the feasibility and effectiveness of the pharmacy-based delivery model, the stakeholders noted that limited research experience among pharmacy providers can result in inconsistent reporting practices and noncompliance with research protocols. To address these challenges, several measures were proposed to ensure the study's success and maintain data integrity. First, the engagement of a research assistant stationed at each pharmacy during the pilot phase was recommended to ensure accurate and high-quality data reporting. Additionally, the establishment of a monitoring team comprising selected stakeholders, including at least one HIV clinician as well as representatives from the PCN and ACPN, was suggested to oversee the study's progress and provide support throughout the research process. The stakeholders suggested that collaborating with the director of pharmaceutical services (DPS) to integrate all pharmacies delivering PrEP services into the State Integrated Supportive Supervision Visits (ISSV) team will be important in the long term. This will help strengthen oversight and quality control. Moreover, they suggested the incorporation of the program's data from participating pharmacies into the Health Management Information System (HMIS) for the private sector currently under development to ensure seamless data integration. Finally, they recommended that the end-of-project results should be disseminated widely and submitted to the Registrar of the PCN, highlighting key policy implications to the Pharmacy Council for review by its governing body.

## Discussion

The collaborative engagement with stakeholders mirrors similar efforts in Kenya, where stakeholder input played a pivotal role in developing a care pathway that successfully integrated PrEP into retail pharmacies [27]. The meeting participants proposed several key recommendations: providing training for pharmacy providers, increasing awareness, safeguarding client confidentiality, building referral networks, and incorporating program data into the Health Management Information System (HMIS). To ensure the success of the pilot study, stakeholders also suggested appointing a research assistant, creating a monitoring team, and submitting the results to the PCN for review.

Retail pharmacies in Nigeria, as in many sub-Saharan African countries, have long been trusted and accessible sources for essential child health medicines and family planning services [28]. This established role positions them well to support the delivery of pharmacy-based PrEP, particularly for at-risk young people, who may encounter stigma or other barriers in accessing traditional healthcare facilities. Compared with clinics, retail pharmacies provide greater convenience, with closer proximity, extended operating hours, and quicker service, making them a more responsive option for clients [29]. Additionally, this approach aligns with global task-shifting trends, where responsibilities typically managed by clinicians are transferred to other healthcare providers, such as pharmacists and community health workers. This shift has expanded coverage and access to essential health services, eases the burden on overstretched healthcare systems, and reduces healthcare costs [30, 31].

This collaborative meeting, involving a broad range of stakeholders, aimed to establish the foundation for the first national framework for pharmacy-based PrEP delivery in Nigeria, effectively bridging the public and private sectors. Stakeholders discussed key challenges and potential solutions for implementing the pharmacy-based PrEP delivery model by adapting the Kenyan model to fit the Nigerian context. In both Kenya and Nigeria, low public awareness of pharmacies as PrEP delivery points was identified as a significant challenge [27]. In Kenya, restrictions on pharmacy advertising for medical services have led to strategies such as word-of-mouth promotion and the discreeting of posters. In contrast, Nigerian stakeholders proposed more proactive measures, such as social media campaigns, community volunteer involvement, and outreach to tertiary institutions. Both countries recognized the need for long-term solutions through national awareness campaigns and policy adjustments. While Kenya focuses on revising advertising restrictions, Nigeria aims to incorporate pharmacy-based PrEP services into the National Health Promotion Policy (NHPP) as part of broader integration into the national health promotion framework. Additionally, stigma surrounding HIV remains a barrier in both countries [32, 33], but the proposed solutions differ slightly. In Kenya, pharmacies were paired with health facilities to alleviate client concerns, whereas Nigerian stakeholders emphasized training pharmacy providers on stigma reduction and ensuring confidentiality through private consultation spaces.

The absence of clear guidelines for HIV testing in pharmacies poses challenges in both Kenya and Nigeria. Both contexts underscore the importance of establishing clear testing protocols and obtaining approval from health authorities to conduct rapid HIV tests in pharmacy settings. The need for pharmacy providers to receive specialized training in PrEP counselling was also recognized, with stakeholders in both countries identifying the lack of training as a barrier to effective service delivery. In Nigeria, stakeholders emphasized integrating PrEP training into mandatory continuing professional development programs for pharmacists. Across both countries, stakeholders highlighted the importance of equipping pharmacy providers with the skills necessary to deliver high-quality, client-centered PrEP counselling.

Pharmacy providers also faced regulatory challenges regarding PrEP prescriptions in both settings. In Kenya, pharmacy providers were initially neither allowed nor trained to prescribe PrEP, necessitating oversight by remote clinicians. Similarly, in Nigeria, aside from remote clinician oversight, a waiver for the pilot study was obtained through ethics clearance since pharmacies do not yet have the authority to initiate PrEP prescriptions. Both countries propose long-term advocacy efforts to change prescribing regulations to allow pharmacies to both prescribe and dispense PrEP.

Operational barriers such as staffing, logistics, and infrastructure were highlighted in both settings. In Nigeria, stakeholders expressed concerns about the lack of time for consultations during peak periods. Their recommended solution was to ask clients to return during off-peak hours for service. To ensure the continuity of service delivery, stakeholders emphasized the importance of multichannel training and engaging multiple staff members within each pharmacy for service offerings. Additionally, while Kenya explored the use of a "PrEP card" system to track clients over time, stakeholders in Nigeria proposed integrating pharmacy-based PrEP delivery services into electronic medical records (EMRs) to facilitate continuity and coordination of care.

The stakeholder meeting using the Nominal Group Technique (NGT) to adapt the Kenyan pharmacy-based PrEP delivery model to the Nigerian context has several limitations worth noting. We relied on a small, selected group of stakeholders, which may not fully represent the diversity of perspectives across Nigeria. Additionally, group dynamics and power imbalances could have influenced the outcomes, leading to potential biases. The time constraints of the NGT also limited in-depth discussions on complex issues, such as regulatory processes needed to enable pharmacies to prescribe PrEP. However, recognizing that adaptation is an ongoing process, we plan to continuously engage relevant stakeholders throughout the pilot implementation. While the meeting generated ideas to address potential challenges, we acknowledge that other practical implementation issues may emerge as we encounter real-world conditions.

## Conclusion

Stakeholders in Nigeria expressed strong support for pharmacy-based PrEP delivery, identifying challenges similar to those seen in Kenya but proposing solutions tailored to Nigeria's healthcare context. Key issues such as demand generation, client assessment, testing, dispensing, and operations were addressed. Drawing on lessons from Kenya, the group developed a model for delivering PrEP through pharmacies to reach at-risk youth in particular. These efforts have laid the groundwork for a refined PrEP framework that is ready for pilot testing and potential nationwide scaling.

## Data Availability

All data generated or analyzed during this study are available upon request.

## Appendix

**Table:** Summary report of stakeholder meeting

Challenges	Potential challenges	Short-term solution	Long-term solution
*Demand generation*			
*Assessment*			
*Testing*			
*Dispensing*			
*Consultation*			
